# Risky sexual behavior among patients on long-term antiretroviral therapy: a prospective cohort study in urban and rural Uganda

**DOI:** 10.1186/s12981-018-0203-1

**Published:** 2018-10-19

**Authors:** Stephen Okoboi, Barbara Castelnuovo, David M. Moore, Joseph Musaazi, Andrew Kambugu, Josephine Birungi, Pontiano Kaleebu, Mastula Nanfuka, Moses R. Kamya, Annelies Van Rie

**Affiliations:** 1 Infectious Diseases Institute, College of Health Sciences, Makerere University, P.O BOX 22418, Kampala, Uganda; 2 Global Health Institute, University of Antwerp, Antwerp, Belgium; 3 Faculty of Medicine and Health Sciences, University of Antwerp, Antwerp, Belgium; 4 British Columbia Centre for Excellence in HIV/AIDS, Vancouver, Canada; 5 University of British Columbia, Vancouver, Canada; 6 The AIDS Support Organization, Kampala, Uganda; 7 Medical Research Council/Uganda Virus Research Institute, Kampala, Uganda; 8 School of Medicine, College of Health Sciences, Makerere University, Kampala, Uganda

**Keywords:** HIV, Antiretroviral therapy, Sexual behavior, Longitudinal cohorts, Uganda

## Abstract

**Background:**

While the effects of initiation of antiretroviral treatment (ART) on risky sexual behavior have been extensively studied, less is known about the long-term changes in risky sexual behavior over time in resource-poor settings.

**Methods:**

We conducted a secondary longitudinal analysis of one rural and one urban cohort of patients who initiated ART in Uganda between April 2004 and July 2007 followed up-to 2016. Data on sexual behavior were collected every 6 months for 3.5 years in individuals on ART ≥ 4 years (baseline) when a behavioral questionnaire was introduced. Risky sexual behavior was defined as sexual intercourse with ≥ 2 partners or inconsistent or no condom use in previous 6 months. We report characteristics overall, and by cohort. We used multivariable generalized estimating equations logistic regression to assess the effects of time on ART on risky sexual behavior.

**Results:**

Of 1012 participants, 402 (39.8%) were urban and 610 (60.2%) were rural residents. Mean age was 42.8 years (SD 8.5). Mean duration of follow-up was 51.3 months (SD 15.3), but longer for urban than rural participants (64.5 vs 36.4 months). Risky sexual behavior declined from 33.1% at baseline to 9.6% after 3.5 years of follow-up in the rural cohort (p ≤ 0.01 for the test of trend) and was unchanged from 9.7% at baseline to 9.9% after 3.5 years in the urban cohort (p = 0.51). Receiving care at a rural clinic (aOR 4.99, 95% CI 3.64–6.84); male gender (aOR 1.66, 95% CI 1.26–2.19) and being younger (aOR 5.60, 95% CI 3.80–8.25 for 18–34 years and aOR 2.34, 95% CI 1.74–3.14 for 35–44 years) were associated with increased odds of risky sexual behavior. Not being married (aOR 0.25; 95% CI 0.19–0.34), and longer time on ART (aOR 0.71 95% CI 0.67–0.76) were associated with reduced odds of risky sex.

**Conclusions:**

We observed a decline in risky sexual behavior in rural people on long-term (≥ 4 years) ART. Rural, male and young individuals had higher odds of self-reported risky sexual behavior. ART programs should continue to emphasize risk reduction practices, especially among people receiving care in rural health facilities, males, younger individuals and those who are married.

## Background

Antiretroviral therapy (ART) transforms HIV infection from a fatal disease to a chronic, manageable condition, increases the quality of life of people living with HIV, greatly improves survival, and reduces the risk of transmission of HIV to a sexual partner [1–3]. Beginning in 2004, ART use in sub-Saharan Africa was dramatically expanded using a public health approach, which led to a rapid expansion of access to treatment in resource-limited settings [1, 2, 4]. In 2017, an estimated 21.7 million people globally were on ART [1]. In Uganda, the number of people on ART is about 750,000 in 2014 [5]. The number of people on ART is likely to further increase following the “recent ‘Treat All’ recommendations by the World Health Organization (WHO) [2, 3]. The educational campaigns around reduced HIV transmission risk with suppressed viral load (Undetectable = Untransmittable UNAIDS message) and reduced HIV related morbidity and mortality may change the sexual behaviour of persons receiving ART [6] as well as HIV negative individuals. Whilst the public discussion of ART prevention benefits has intensified in the framework of the WHO ‘Treat All’ strategy, one of the issues voiced is that this might result in increased sexual risk-taking [6–10].

Prior studies have evaluated the association between ART and risky sexual behavior in the first one to 3 years following ART initiation. Prospective cohort studies in South Africa and Uganda found a decrease in risky sexual behavior. In Uganda, risky sexual behavior in men decreased from 16.2% before ART to 4.3% at 12 months after ART initiation [11]. In South Africa, risky sexual behavior decreased 12 months after ART initiation compared to pre-ART (aOR 0.86, 95% CI 0.78–0.95) [12]. In a randomized clinical trial of three different ART monitoring strategies in rural Uganda, risky sexual activity decreased from 22% at ART initiation to 14% at 36 months of ART follow up [13, 14]. In contrast, cross-sectional studies from Togo, Uganda and Cote d’Ivoire found higher rates of participants practicing risky sexual behavior after short durations on ART [15–19]. These conflicting results highlight the complexity of examining the association of ART and risky sexual behavior and its variations in different settings, study participants and treatment program characteristics. Two meta-analyses summarized the association between ART and sexual risk behavior. A meta-analysis of 8 longitudinal studies and 6 cross-sectional studies of the sexual behavior of patients on ART in Sub Saharan Africa (SSA) found a reduction in risky sexual behavior as compared to pre-ART (pooled effect estimate OR 0.47, 95% CI 0.25–0.89 for multiple sexual partners). Furthermore, people on ART were 55% more likely to have protected sex with a partner who was HIV negative or of unknown HIV status as compared to an HIV infected partner [20]. Another meta-analysis of 55 prospective and cross-sectional studies in SSA also found lower sexual risk-taking (OR 0.7; 95% CI 0.62–0.81) among participants on ART compared with those not on ART [21].

To date, most studies examined the effect of ART on sexual risk behavior in the first year(s) of ART; and mainly compared risky sexual behavior before and after ART initiation. Data on the risky sexual behavior among adults on long-term (≥ 4 years) ART in sub-Saharan Africa is limited. Using data from two clinical cohorts, we evaluated long-term changes in risky sexual behavior among people receiving ART in urban and rural Uganda.

## Methods

### Study design

This was a secondary analysis of two longitudinal cohorts of individuals on long-term ART: the ART cohort of The Infectious Diseases Institute (IDI), College of Health Sciences at Makerere University in Kampala, Uganda [22] and the ART cohort of The AIDS Support Organization, (TASO) in Jinja, Uganda [23]. The two cohorts had a 2-year difference in ART initiation timelines and this limited direct comparison.

### Study setting

#### The IDI urban ART cohort in Kampala

The adult clinic of the Infectious Diseases Institute (IDI) is a Centre of excellence for HIV care and treatment located in the Mulago Teaching Hospital in Kampala-Uganda. IDI is a large outpatient clinic with over 30,000 patients ever enrolled and cares for people living in five Kampala municipalities. ART is provided free of charge, initially through the Global Fund and later mainly through the US President’s Emergency Plan for AIDS Relief (PEPFAR).

Following written informed consent, adults (age ≥ 18 years) starting ART between April 2004 and April 2005 were enrolled and followed up (hereafter referred to as the IDI urban cohort). ART was started in patients with WHO Stage 4 or CD4 count ≤ 200 cells/ml, (according to national treatment guidelines implemented at that time), and consisted of stavudine (weight-adjusted and its use discontinued in 2013 in Uganda), lamivudine and nevirapine (fixed-dose combination) or zidovudine, lamivudine (fixed-dose combination) and efavirenz. A study doctor and adherence counsellor evaluated patients at study enrolment and every 6 months thereafter, while they attended the general clinic for monthly ART medication refills [22]. At each study visit, a physical examination was performed and information was recorded about HIV status of the partner(s), social support, and sexual history in the past month including promotion of condom use, adherence using visual analogue scale, and reasons for non-adherence. ART was monitored every 6 months through viral load (VL) testing. Participants with a VL ≥ 1000 copies/ml were offered enhanced adherence counselling and support including a face-to-face session to discuss the implications of unsuppressed viral load (VL). Adherence strategies used by the patient were reviewed, and an adherence action plan developed. Patients with two consecutive viral loads ≥ 1000 copies/ml were considered eligible for a second-line ART regimen.

#### The TASO rural ART cohort in Jinja

The AIDS Support Organization is one of the first and largest non-governmental organizations in SSA, providing treatment to over 98,000 patients in Uganda. Since 2004, TASO Jinja, one of 11 TASO care facilities in Uganda, has cared for people living with HIV in the Jinja district within a radius of 75 km and currently provides ART to over 5000 people.

In June 2012, TASO Jinja initiated a prospective longitudinal cohort (Long-term Outcome on ART study) among patients who initiated ART between 2004 and 2007 using the WHO Stage 4 or CD4 count ≤ 200 cells/ml, (according to national treatment guidelines implemented at that time), and consisted of stavudine (weight-adjusted and its use discontinued in 2013 in Uganda). The Long-Term Outcomes on ART study recruited patients receiving first-line ART for a minimum of 4 years (hereafter referred to as the TASO rural cohort) [23, 24]. Between July 1, 2012, and December 31, 2013, all individuals receiving first-line ART for at least 4 years were eligible to participate in the cohort study and were included after written informed consent. Participants continued to receive adherence counselling and condom use from TASO counsellors and peer educators mostly in groups as part of routine care. From August 2014 until January 2015, enrollment was restricted to patients already on ART for at least 4 years with CD4 cell count ≤ 450 cells/mm^3^ in order to over-sample for participants who may have virologic failure. All participants continued to receive routine comprehensive HIV care from the TASO service providers. Routine VL testing was not available at TASO Jinja at the time of the study but all participants received a VL test at enrollment. Participants with a VL ≥ 1000 copies/ml were offered enhanced adherence counselling and support including a face-to-face session to discuss the implications of unsuppressed VL, assessment of the adherence strategies used by the patient, and development of an adherence action plan. VL was re-assessed after 3 months.

Patients were followed up for additional 3.5 years. Every 6 months, cohort participants completed an interviewer-administered standardized questionnaire (adapted from the HAARP study) [24] to collect behavioral and treatment outcomes. Laboratory monitoring included CD4 counts and VL every 6 months. CD4 counts were performed at the Jinja referral hospital regional laboratory. Plasma samples were stored in liquid nitrogen and shipped to the MRC/UVRI laboratory in Entebbe, Uganda for VL testing and resistance testing.

### Data collection and management

The IDI urban cohort data were directly collected into an electronic behavioral medical record by study counsellors who provided risk reduction counselling including condom use promotion and periodically validated by a senior data manager. The TASO rural cohort, data were collected by the research assistants (counsellors) using an interview administered behavioral questionnaire but they did not provide counselling on condom use promotion. Data were double entered using Epi-Info and imported into Access for data management and storage. Laboratory test results were transmitted electronically from the MRC laboratory to the data Centre in TASO Jinja, manually entered in the study database.

For this study, data extracted from TASO and IDI database included socio-demographic information (age, gender, educational level, employment, and marital status), ART start date and regimen, clinical data collected at enrollment, including WHO clinical stage, CD4 cell counts, viral load and sexual behavior (number of partners in past 6 months and consistency of condom use) and adherence data at enrolment and subsequent clinic visits. Risky sexual behavior was defined as sexual intercourse with ≥ 2 partners or sexual intercourse with 1 partner and inconsistent condom use.

### Statistical analysis

For this analysis, baseline was defined as the time of enrollment into the cohort which occurred after ≥ 4 year on ART (rural cohort) or at the 4-year on ART visit (urban cohort). For all patients included in the cohort, data was extracted for an additional 3.5 years after this baseline visit. All participants were followed until death, loss to follow up, transfer out, withdrawal of consent, or 3.5 years after the “baseline” visit.

We described participants characteristics at baseline overall, by gender and cohort (urban/IDI and rural/TASO) using means and frequencies. Continuous variables were compared using an unpaired t-test or Wilcoxon rank sum test if not normally distributed. Pearson Chi-square was used to compare categorical variables.

The main exposure of interest was time on ART (coded as 6-month periods). Other covariates of interest were the site, age, CD4 cell count, viral load, gender, education level, marital status, occupation, and ART regimen at baseline and calendar year of ART initiation. In order to examine the effect of long-term ART on sexual behaviors, separate analyses were performed. At baseline, we performed multivariable logistic regression and included variables in the initial model based on prior knowledge or association of the selected covariates with risky sexual behavior in bivariable analysis. Using a backwards elimination procedure, we started with all covariates in the model and then stepwise removed the covariate with the largest p-value until all the remaining covariates had a p-value less than 0.10 for the association with the outcome of interest.

To model the association between the binary outcome (risky sexual behavior) and the exposure of time on ART, we used a Generalized Estimating Equations (GEE) logistic regression model, with a logit link function to model the association between the binary outcome (risky sexual behavior) and the exposure of time on ART. Using a GEE logistic regression, odds ratios (ORs) were derived that took into account the repeated measures in individual participants and missing data in response variables, using robust standard errors to account for within-subject correlation. Co-variates with a p-value less than 0.10 for the association with the outcome of interest was included in the final model. Data were analyzed using STATA version 15 (StataCorp, College Station, TX).

### Effect modifiers exploratory analysis

We assessed for effect modification by the following variables: gender and sexual partners, marital status and sexual partners, current age group and sexual partners. Interaction terms with a p-value < 0.05 were considered statistically significant.

## Results

Overall, 1012 individuals receiving ART for ≥ 4 years were included in the analysis. Of these 402 (39.8%) were from the urban (IDI) cohort and 610 (60.2%) from the rural (TASO) cohort. Most patients were female (69.6% urban vs 75.7% rural cohort) and about half (53.3% urban vs 54.4% rural) of all patients were not married. At baseline, the mean age was 42.8 years (SD = 8.5), with rural patients being older than urban patients (mean age 44.6 vs 39.8 years; p-value 0.05) (Table 1). Slightly over a half (53.5%) of the urban but only 35.3% of the rural participants attained secondary education or higher (p-value < 0.01). At baseline, the most common ART regimen was zidovudine, lamivudine and nevirapine in the rural cohort and a tenofovir-based regimen in the urban cohort. At baseline, almost all participants (~ 90% in both cohorts) had a CD4 > 200 cells/mm^3^, but the mean CD4 count was lower in the urban cohort (mean CD4 cell count 426 cells/mm^3^ and 564 cells/mm^3^ for urban cohorts and rural, respectively).

**Table 1:** Baseline characteristics of the cohort participants

Variable	N	Urban (IDI)	N	Rural (TASO)
		N (%)		N (%)
Baseline age in years	402		610	
18–34		118 (29.3)		52 (8.5)
35–44		180 (44.8)		274 (44.9)
> 45		104 (25.9)		284 (46.6)
Gender				
Female		280 (69.6)		462 (75.7)
Male		122 (30.4)		148 (24.3)
Marital status				
Married/cohabiting		187 (46.5)		278 (46.6)
Not married		215 (53.5)		332 (54.4)
Educational level				
No formal education		15 (3.7)		110 (18.0)
Primary level		173 (43.0)		285 (46.7)
≥ Secondary level		214 (53.3)		215 (35.3)
Employment				
Formal employment		178 (44.3)		105 (17.6)
No formal employment		224 (55.7)		491 (82.4)
Baseline ART regimen				
ZDV 3TC EFV		123 (33.5)		113 (18.5)
D4T 3TC NVP		66 (18.0)		0
ZDV 3TC NVP		0		356 (58.3)
TDF based regimen		177 (48.5)		141 (23.0)
Mean time on ART		8.7 (SD 1.2)		7.0 (SD 0.3)
Baseline viral load				
Mean log 10 viral load copies/ml		2.65 (SD 0.34)		1.91 (SD 1.27)
< 1000 cells/ml		385 (96)		501 (82.1)
≥ 1000 cells/ml		16 (4.0)		109 (17.9)
Baseline CD4 cell/mm^3^				
Mean CD4		426(SD 194.4)		564.3 (284.2)
< 200 cells/mm^3^		43 (10.7)		54 (8.85)
200–499 cells/mm^3^		237 (60.0)		213 (34.9)
> 499 cells/mm^3^		122 (30.4)		340 (55.7)
WHO staging				
Stage 1		2 (0.5)		25 (4.2)
Stage 2		51(12.7)		243 (41.3)
Stage 3		232 (57.7)		205 (34.8)
Stage 4		117 (29.1)		116 (19.7)

Some percentages do not add up to 100% because of missing data

### Follow up

A total of 941/1012 (93%) participants completed 3.5 years of follow up after baseline. The mean duration of follow-up was 51.3 months (SD 15.3), but longer for urban than rural participants (64.5 vs 36.4 months). After enrollment, 38 (3.8%) participants died 21 (5.2%) urban and 17 (2.9%) rural participants. At 3.5 years of follow up, few (3.3%) patients were lost to follow up or transferred out (16 urban and 17 rural participants), and only 15 (1.5%, all urban) participants withdrew study consent.

### Risky sexual behavior at baseline (≥ 4 years visit on ART)

Overall, 241 (24.0%) cohort participants reported having engaged in risky sexual behavior at baseline. The type of risky sexual behavior was predominantly inconsistent or no condom use while engaged in a sexual relationship with a single partner (87.1%, 210/241). Engagement in sexual relationships with ≥ 2 partners was less prevalent (12.9%, 32/241). Participants reporting risky sexual behavior at baseline were more likely to be aged 18–34 and 35–44 years, female, married, and rural residency (p < 0.01 for all) (Table 2).

**Table 2:** Baseline participants risky sexual behavior comparison

Variable	N	Non risky (N = 769)	Risky sex (N = 241)
		N (%)	N (%)
Site	1010		
Urban		363 (47.2)	39 (16.2)
Rural		406 (52.8)	202 (83.8)
Age in years	1010		
18–34		131 (17.1)	38 (15.8)
35–44		331 (43.0)	127 (51.0)
≥ 45		307 (39.9)	80 (33.2)
Gender	1010		
Female		593 (77.0)	147 (61.0)
Male		176 (23.0)	94 (39.0)
Marital status	1010		
Married/cohabiting		265 (34.5)	200 (90.0)
Not married		504 (65.5)	41 (17.0)
Educational level	1010		
No formal education		97 (12.6)	27 (11.2)
Primary Level		343 (44.6)	114 (47.3)
≥ Secondary level		326 (42.8)	100 (41.5)
Employment	996		
Formal employment		231 (30.1)	57 (22.0)
No formal employment		529 (69.9)	184 (80.0)
Baseline viral load	1009		
< 1000 cells/ml		678 (88.3)	207 (85.9)
≥ 1000 cells/ml		90 (11.7)	34 (14.1)
Baseline CD4	1010		
< 200 cells/mm^3^		71 (9.2)	29 (12.0)
200–499 cells/mm^3^		355 (46.0)	95 (39.4)
> 499 cells/mm^3^		345 (44.8)	117 (48.6)

In univariate logistic regression analysis, rural residence (OR 4.61, 95% CI 3.18–6.68), being male (OR 2.16, 95% CI 1.58–2.94), formal employment (OR 1.54, 95% CI 1.09–2.17), viral load > 1000 copies/ml (OR 1.22, 95% CI 0.80–1.87), and being younger [18–34 years (OR 1.11, 95% CI 0.72–1.72) and 35–44 years (OR 1.43, 95% CI 1.04–1.97) compared with age > 45 years] were associated with an increased odds of risky sexual behavior. Not being married was associated with lower odds of risky sexual behavior (OR 0.11, 95% CI 0.07–0.55).

In multivariable logistic analysis, rural residence (adjusted odds ratio [aOR] 9.54, 95% CI 5.83–15.61) being male (aOR 1.51, 95% CI 1.02–2.31) and younger age, (aOR 3.41, 95% CI 1.87–6.21 for 18–34 years and aOR 2.12, 95% CI 1.40–3.21 for 35–44 years) remained strongly associated with increased odds of risky sexual behavior. Not being married remained associated with lower odds of risky sexual behavior (aOR 0.10; 95% CI 0.06–0.14) (Table 3).

**Table 3:** Risky sexual behavior and associations with ART among cohort participants

Variable	Baseline (≥ 4 years risky sexual behavior)		GEE logistic analysis	
	Univariate logistic	Multivariate logistic	Un adjusted GEE	Adjusted GEE
	OR (95% CI)	OR (95% CI)	OR (95% CI)	OR (95% CI
Site				
Urban	Ref	Ref	Ref	Ref
Rural	4.61 (3.18–6.68)	9.54 (5.83–15.61)	2.06 (1.58–2.70)	4.99 (3.64–6.83)
Baseline age in years				
18–34	1.11 (0.72–1.72)	3.41 (1.87–6.21)	2.58 (1.83–3.65)	5.60 (3.80–8.25)
35–44	1.43 (1.04–1.97)	2.12 (1.40–3.21)	1.86 (1.39–2.49)	2.34 (1.74–3.14)
> 45	Ref	Ref	Ref	Ref
Gender				
Female	Ref	Ref	Ref	Ref
Male	2.16 (1.58–2.94)	1.51 (1.0–2.31)	1.82 (1.41–2.35)	1.66 (1.26–2.19)
Marital status				
Married/cohabiting	Ref	Ref	Ref	Ref
Not married	0.11(0.07–0.55)	0.09 (0.06–0.14)	0.26 (0.20–0.34)	0.25 (0.19–0.34)
Educational level				
No formal education	Ref		Ref	
Primary level	1.20 (0.74–1.94)		1.30 (0.85–1.99)	
≥ Secondary level	1.10 (0.68–1.79)		1.45 (0.95–2.22)	
Employment				
No formal employment	Ref	Ref	Ref	
Formal employment	1.54 (1.09–2.17)	0.93 (0.60–1.44)	1.18 (0.89–1.55)	
Viral load				
< 1000 cells/ml	Ref	Ref	Ref	Ref
≥ 1000 cells/ml	1.22 (0.80–1.87)	0.52 (0.23–0.96)	1.26 (0.88–1.79)	0.57 (0.37–0.87)
Baseline CD4				
<200 cells/mm^3^	Ref	Ref	Ref	Ref
200–499 cells/mm^3^	0.66 (0.49–1.07	0.57 (0.30–1.10	0.67 (0.45–0.99)	0.59 (0.38–0.90)
>499 cells/mm^3^	0.51 (0.51–1.34)	0.63 (0.32–1.23)	0.62 (0.42–0.92)	0.52 (0.33–0.84)
Time on ART			0.76 (0.72–0.80)	0.71 (0.67–0.76)
TASO cohort sub-analysis				
No. of life time partners	1.00 (1.10–1.02)		1.01 (1.00–1.02)	
Age of first sex	0.98 (0.92–1.06)		1.02 (0.97–1.07)	

### Risky sexual behavior over time

**Fig. 1:**
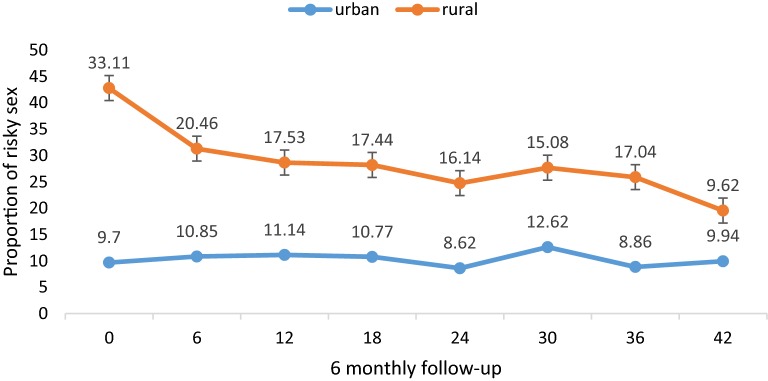
Trends in risky sexual behavior among urban and rural cohort participants after additional 3.5 years of follow up

The proportion of rural patients self-reporting risky sexual behavior declined from 33.1% at enrollment to 9.6% at 3.5 additional years of follow up (p-value for trend < 0.01). In contrast, the proportion of urban patients self-reporting risky sexual behavior remained stable: 9.7% at enrollment, 11.2% at 1 year later, 12.6% at 2.5 years later and 9.9% at 3.5 years later of follow up (p value for trend 0.71) (Fig. 1).

In the multivariable (GEE) logistic regression model, taking repeated measures over time into account, rural residence (aOR 4.99, 95% CI 3.64–6.83); male gender (aOR 1.66, 95% CI 1.26–2.19) and younger age; (aOR 5.60 95% CI 3.80–8.25 for 18–34 years and aOR 2.34 95% CI 1.74–3.14 for 35–44 years) were associated with increased odds of risky sexual behavior. Not being married (aOR 0.25, 95% CI 0.19–0.34) and time on ART (aOR 0.71 per year, 95% CI 0.67–0.66), baseline CD4 cell/mm^3^ (aOR 0.59, 95% CI 0.38–0.90 for 200–499 cells/mm^3^ and aOR 0.52 95% CI 0.33–0.84 for > 499 cells/mm^3^) were associated with reduced odds of risky sexual behavior (Table 3).

Educational level, type of employment, the age of first sexual intercourse (aOR 112; 95% CI 0.80–1.78) and number of lifetime sexual partners (aOR 0.95; 95% CI 0.90–1.01) were not associated with risky sexual behavior.

## Discussion

Among people on ART for ≥ 4 years we observed that rural residence was associated with increased odds of self-reported risky sexual behavior. Risky sexual behavior was predominately due to poor condom use and much less due to multiple sexual partners. Risky sexual behavior increased with younger age, with a 2.12-fold increased odds among 35–44 years and 3.41-fold higher odds among 18–34 years compared to individuals age 45 years or older. The odds of risky sexual behavior decreased with time of follow up on ART (aOR 0.71), but this decrease over time was only present in the rural cohort. When taking the sexual behavior over the entire period of long-term ART into account, the odds of risky sexual behavior was higher among male compared to female participants (aOR 1.66), while a higher CD4 count (aOR 0.52–0.59) and those who failed to achieve viral load suppression (aOR 0.52) at baseline was associated with a lower odds of risky sexual behavior.

Comparison with other studies in sub-Saharan Africa is difficult as longitudinal data on risky sexual behaviors among people on long-term ART is limited. Our findings do suggest that the reduction in risky sexual behavior observed in the first year(s) of ART in several studies in sub-Saharan Africa [9, 12, 14, 20, 21, 25–31] does not revert into increased risky sexual behavior over time, but instead either stabilizes at a relatively low rate (as observed in the urban cohort) or further decreases over time (as observed in the rural cohort). Even though the risk reduced or stabilized over time, the continued risk-taking emphasizes that it remains important to focus on individual risk reduction practices such as consistent condom use even among patients who are already several years on ART.

We found that rural residence, male gender, and younger age were associated with increased odds of risky sexual behavior, similar to what was observed in sub-Saharan Africa studies of individuals during the first one to 4 years of ART [11–14, 21, 27–29]. For example, in a South African cohort study, people receiving ART at urban and rural health care facilities found younger age, being married and male gender was associated with risky sexual behavior [12]. Our finding of increased risky sexual behavior among males and married is also similar to Ugandan studies that found, among those on ART for one to 4 years, higher or even increased risky sexual behavior among men and those that are married were observed [11, 13, 14, 27, 30]. The increased risky sexual behavior observed among men could be because of the gender and power dynamics, where men are the major decision-makers in sexual issues in Africa. Among the married, the increased risk of sexual behavior is attributed to the desire to have children, fear of HIV status disclosure to the sexual partner(s), and the beliefs that condom use is not necessary for HIV positive couples and reduces sexual satisfaction. The difference in risky sexual behavior among people receiving care at urban and rural facilities could be due to differences in rural vs urban environment with urban participants having greater exposure to HIV prevention messaging and more likely to be of a higher socioeconomic status. Furthermore, condom promotion was mainly done to urban cohort participants.

The reduced odds of risky sexual behavior among participants with higher CD4 cell/mm^3^ and suppressed VL at baseline is similar to the findings of other studies in Uganda [11, 13, 14, 27, 30].

Our study had several strengths, including its prospective design, the inclusion of a cohort in both a rural and urban setting, and the large sample size which allowed us to assess and identify factors that are independently associated with risky sexual behavior. Several limitations need to be taken into account when interpreting the results. First, the two cohorts had a 2-year difference in ART initiation timelines and baseline visit included participants who have been on ART for 4 years in the urban cohort or ≥ 4 years in the rural cohort, complicating straightforward comparison between these two cohorts. Second, only patients from HIV centers of excellence were included, which may not be representative for smaller centers or primary care settings. Furthermore, at the IDI (urban cohort), patients were asked about sexual behavior from ART initiation by the counsellors which were providing risk-reduction counselling, which may lead to patients being less likely to report risky sexual behavior due to social desirability bias. However, the study team was trained to ask questions in a professional and non-judgmental approach to minimize any social desirability. Third, patients included in the study initiated ART based on CD4 eligibility criteria hence our results may not be generalizable to people receiving long term–term ART under the current WHO ‘treat all’ strategy. Future studies should monitor trends of risky sexual behavior in high-risk populations on long-term ART and whether low-risk sexual behavior also occurs among patients on long-term ART in the ‘treat all’ era.

## Conclusions

We observed a decline in risky sexual behavior in rural people on long-term (≥ 4 years) ART. Rural, male and young individuals had higher odds of self-reported risky sexual behavior. ART programs should continue to emphasize risk reduction practices, especially among people receiving care in rural health facilities, males, younger individuals and those who are married.

## Abbreviations

ART: antiretroviral therapy
WHO: World Health Organization
TASO: The AIDS Support Organization
IDI: Infectious Diseases Institute
MRC: Medical Research Council
